# Testing social learning of anti-predator responses in juvenile jackdaws: the importance of accounting for levels of agitation

**DOI:** 10.1098/rsos.171571

**Published:** 2018-01-24

**Authors:** Guillam E. McIvor, Victoria E. Lee, Alex Thornton

**Affiliations:** Centre for Ecology and Conservation, University of Exeter, Penryn, UK

**Keywords:** predator recognition, mobbing, corvids, captivity, habituation, personality

## Abstract

Social learning is often assumed to help young animals respond appropriately to potential threats in the environment. We brought wild, juvenile jackdaws briefly into captivity to test whether short exposures to conspecific vocalizations are sufficient to promote anti-predator learning. Individuals were presented with one of two models—a stuffed fox representing a genuine threat, or a toy elephant simulating a novel predator. Following an initial baseline presentation, juveniles were trained by pairing models with either adult mobbing calls, indicating danger, or contact calls suggesting no danger. In a final test phase with no playbacks, birds appeared to have habituated to the elephant, regardless of training, but responses to the fox remained high throughout, suggesting juveniles already recognized it as a predator before the experiment began. Training with mobbing calls did seem to generate elevated escape responses, but this was likely to be a carry-over effect of the playback in the previous trial. Overall, we found little evidence for social learning. Instead, individuals' responses were mainly driven by their level of agitation immediately preceding each presentation. These results highlight the importance of accounting for agitation in studies of anti-predator learning, and whenever animals are held in captivity for short periods.

## 1. Introduction

The ability to recognize and respond appropriately towards predators is a critical component of fitness. Many young animals appear to recognize predators as being dangerous on their first encounter [1–3], be this through genetically determined responses [4] or through information acquired during development [5,6]. For many species, however, learning during early life plays a major role in the development of predator recognition [7,8]. Learned predator recognition is likely to be particularly advantageous in highly variable or heterogeneous environments, where predator assemblages and predation risk can vary spatially and/or temporally. Animals may learn about predators and potential sources of danger through personal experience of being chased, by observing predators attacking conspecifics [9,10] or through the anti-predator signals and cues of conspecifics alone (reviewed in [11]). In aquatic environments learning about danger via social cues often involves the learned association of chemosensory information, such as the pairing of the chemical distress signals of an attacked conspecific with the scent of the predator [12,13]. By contrast, in terrestrial systems information about predators is commonly conveyed by visual and acoustic means [11]. Vocalizations made in response to predators can act as a warning of danger, eliciting flight responses, but many species also produce distinctive ‘mobbing’ alarm calls when a predator is spotted [14]. These calls can encode information about the nature of the threat [15], triggering responses in both hetero- and conspecifics [16,17] that may either flee or may join the instigator in mobbing the target in an attempt to drive it away from the area [18].

A large body of experimental evidence shows that mobbing vocalizations can also help naive individuals to learn socially about danger. In a pioneering experiment, Curio *et al*. [19] instigated a mobbing response in observer blackbirds (*Turdus merula*) towards a stimulus that they had previously treated as being harmless (a model friarbird, *Philemon corniculatus* or a plastic bottle), by allowing the observer to witness a demonstrator mobbing the model. The demonstrator was in fact mobbing a model owl that was hidden to the observer, but this learned association between the demonstrator's mobbing response and the (harmless) stimulus resulted in the transmission of mobbing responses along a chain of six individuals by using the observer as the demonstrator in each subsequent trial. While the great majority of research has been conducted in controlled laboratory settings, such cultural transmission of predator recognition has since been documented in the wild. For instance, American crows (*Corvus brachyrhynchos*) with no experience of being trapped or observing trapping socially learned to mob researchers who returned to the area wearing the same mask that had been worn when individuals from the previous generation of crows had been captured [20].

In recent decades a number of experiments have successfully trained naive individuals about predators or brood parasites through pairing model predator or brood parasite presentations with a training stimulus. This stimulus was either a live conspecific demonstrator engaging in mobbing [19,21,22], mobbing calls paired with mounts of conspecifics [23], or mobbing calls alone [18,21,24], but in all cases the training phase lasted between 2 and 5 min. While such prolonged learning opportunities may reflect cases in which mobbing continues until the target moves away or the mobbing group loses interest [25–27], in many instances exposure to conspecifics' anti-predator responses are fleeting. For example, many predators are highly mobile, actively hunt their prey using the element of surprise and leave the area rapidly after an unsuccessful hunt (e.g. [28,29]), providing prey species with only brief but vital opportunities for learning from conspecifics’ responses. We tested whether a short, eight-second exposure to conspecific mobbing calls might be sufficient to train individuals to respond fearfully to a novel predator.

We conducted our study on wild jackdaws (*Corvus monedula*), highly social members of the corvid family. Jackdaws breed colonially, and short bursts of scolding calls are commonly heard in colonies in response to passing predators (G. E. McIvor 2014, personal observation). Jackdaw nestlings fledge from cavities from 30 days after hatching [30], and are dependent on their parents for up to 6 weeks thereafter [31,32]. In response to predators, adult jackdaws produce distinctive anti-predator mobbing calls known as scold calls. Scold calls can be emitted singly to act as a warning of danger (e.g. if a predator is passing by), or repeated to recruit others to mob. These calls are likely to play a key role in helping young jackdaws learn about danger. Indeed, Lorenz [7] suggested, based on his observations of hand-reared jackdaws, that predator recognition is socially learned in this species, though this remains to be tested.

To control individuals' exposure to experimental stimuli and allow comparisons of their responses before and after exposure to social information, we brought juveniles temporarily into captivity for testing. This represents a compromise between testing in laboratory conditions, where the proximity of test stimuli and absence of distractions may artificially enhance social learning [33], and field experiments on unconstrained individuals where it can be extremely difficult to ensure that subjects attend to the relevant stimuli over repeated controlled presentations. Free-living juveniles were captured for the experiment 2–6 weeks after fledging using walk-in traps. Captured birds were transferred to a nearby aviary, and after an acclimatization period given a series of presentations of one of two models—a stuffed fox to represent a genuine threat, or a toy elephant that simulated a novel predator. The first presentation that each bird received was made with no accompanying playback, to gain a measure of their baseline response to the model stimulus. In conjunction with the second time the model was presented, the birds also received an 8 s playback of the calls of colony members—either scold calls that suggested danger, or contact calls that suggested no danger. Such brief calling events are common in jackdaw colonies. To test whether the scold/contact call playback in the training phase influenced the subsequent response of individuals towards the model, we presented the model a final time with no accompanying playback. We predicted that birds shown the elephant with scold calls would show a greater escape response to the model than those shown the elephant with contact calls, and would also direct scold calls at the model. By contrast, we expected the jackdaws to already recognize foxes as a threat and thus show an equally high escape response to the fox model regardless of which playback they had received. Finally, we predicted that the response of birds in the elephant-scold group would match the escape response level of those birds shown the fox model.

## 2. Material and methods

### 2.1. Subjects and housing

The experiment was carried out over 14 days in July 2015. Forty-eight juvenile jackdaws were captured using a passive walk-in trap baited with bread and oats. Half of the birds used in the experiment had been ringed as chicks at our study site and had a known fledge date. On average these birds fledged from the nest 31 days before capture (range = 19 to 43 days). As breeding is highly synchronous across our jackdaw population (greater than 90% of all nests fledge within a 14-day period; G. E. McIvor, V. E. Lee, A. Thornton 2015, unpublished data), the 24 birds whose fledging date was unknown were likely to have been at large in the environment for a similar length of time and have had a similar likelihood of having encountered predators as the birds with known fledging dates.

Once captured, individuals were removed from the walk-in trap and transferred to a separate, temporary aviary in a field 100 m away. Although we cannot rule out the possibility that jackdaws flying past may have observed experimental presentations to other individuals before they themselves received them, this is very unlikely given that models were only displayed for a few seconds during presentations before being returned to a concealed position. The aviary consisted of a wooden frame (2 × 3 × 1.8 m) covered by game-bird netting, which allowed the bird to see in all directions. A diagram of the experimental arena is provided in the electronic supplementary material. Half of the roof was covered in wooden boards to provide shade, and two screens were provided at the back of the aviary behind which the bird could hide. Branches were provided as perches in each corner, and a central beam ran across the middle of the arena that could also be used as a perch, as could the ledges than ran around the outside of the arena. All birds were provided with a dish containing food (rolled oats) and water. Birds were typically kept in the aviary for 25 to 35 min, after which they were released.

### 2.2. Experimental procedure

Following transfer from the walk-in trap to the experimental aviary, each jackdaw was given 10 to 15 min to calm down and acclimatize to its surroundings before the experiment commenced. This start time was consistent with the exception of two of the 48 trials, where the birds received longer periods because of farm machinery moving nearby (mean duration of acclimatization period (s.e.) = 12.75 (0.51) min; range: 10–32 min). The 10 to 15 min period was considered to be an acceptable compromise between the need for time to acclimatize to the aviary and the ethical requirement of minimizing the total length of time that juvenile birds were separated from their parents, upon whom they were still partially dependent. Birds were shown one of two models, in a series of three separate presentations: (1) *baseline*, (2) *training* and (3) *test*. Twenty-four birds were shown a toy elephant to simulate a novel predator. This stimulus was deliberately chosen so as to avoid any potential resemblance to any animal the jackdaws may have encountered previously. To provide an ecologically relevant comparison, the other 24 birds were shown a taxidermy model of a red fox (*Vulpes vulpes*), a predator that is common around our study site and is frequently mobbed by jackdaws when encountered. The elephant model was of roughly equivalent height and length as the fox model. Birds were assigned to their model group at random, and received three presentations of the same model. The model was located in a hide 10 m from the front of the aviary, and was mounted onto a skateboard to allow it to be smoothly moved in and out from the concealed position. Stones were piled in front where the models emerged so that the birds would only see the model, and not the skateboard. The skateboard's movement was controlled by an experimenter inside an adjacent hide by way of a connecting broom-handle (see the electronic supplementary material, figure S1).

Presentation 1 was intended to measure the *baseline* response of the bird to the model, and involved the model being rolled from the concealed position and sitting stationary in the open for 8–10 s before returning to cover, with no accompanying playback. Five minutes after presentation 1, the birds were shown the same model again in presentation 2, the *training* phase, where the model was accompanied by a playback. The playback contained either contact calls that would suggest there was no danger posed by the model, or scold calls that would suggest that the model was dangerous. Both the model and playback type to which the birds were assigned were allocated at random, but with a limit of 12 individuals to each combination of model and playback type. The specific playback track that the bird received during the *training* presentation was also allocated at random, from a choice of six playback tracks for both the scold and contact call groups.

All calls used for playbacks were collected using Olympus LS-100 digital recorders (Olympus Corporation, Tokyo, Japan), used in conjunction with two types of microphone. Contact calls were obtained by recording calls between pairs at nest boxes using AKG-C417PP lapel microphones (AKG Acoustics, Vienna, Austria). Scold calls were collected using Sennheiser ME66 shotgun microphones (Sennheiser Electronic GMBH & Co. KG, Wedemark, Germany) with a Reinhardt windshield when nests were being visited by researchers. There is no evidence that the scold calls of jackdaws are predator specific, and previous experiments at our study sites show that playbacks of scold calls recorded in response to humans elicit collective anti-predator responses as if a predator were present [34]. In our recordings, the identity of all callers was known, and only calls from adult individuals nesting in the colony where the experiment was carried out were used. Callers were not related to test subjects, and the parentage of all marked juveniles was checked prior to the trials to ensure they had not been allocated a playback track that contained the calls of a parent. Each playback contained the calls of four birds, and each bird contributed three calls each. Playback tracks were prepared using the open source audio editing software Audacity (www.audacityteam.org), with normalized amplitude across all tracks. The playbacks were made through three separate FoxPro Fury (FOXPRO Inc., Lewistown, PA, USA) speakers, to simulate a natural bout of calling by a group of jackdaws. Speakers were set to volume level 18, simulating the amplitude of a calling bout 10 m away (c. 72 dB) as measured using a Voltcraft SL-100 sound level meter. The three speakers were set to the same operating channel to allow them to be controlled by a single handset. They were arrayed in an arc around the hide from which the model was presented, and each speaker contained a different track loaded in each position on their memory. This meant that all three speakers would play a different track of four calls simultaneously over an 8 s period. This produced a playback of 12 calls in total, containing calls from three individual birds, with each individual contributing four calls each (see the electronic supplementary material, S1). The playback slot was assigned at random for each bird. Each of the three tracks that occupied a memory slot had been prepared simultaneously in Audacity, and this ensured that they each achieved an identical effect. Playbacks were arranged so that the time between calls on the three synchronized tracks decreased as the playback continued, simulating the build-up of calls that occurs naturally during social chatter(contact call group) or recruitment to a mobbing event (scold call group).

Five minutes after the end of presentation 2 the birds received the third and final *test* presentation. The procedure was identical to presentation 1, and served to test whether the responses of the birds changed as a result of the playbacks they heard in presentation 2. Five minutes after the end of presentation 3, the birds were removed from the aviary and released. The birds were filmed for the full time spent in the aviary using a Panasonic HC-X920 camera (Panasonic Corporation, Osaka, Japan).

### 2.3. Video and data analysis

Videos were coded using Noldus Observer XT12 (Noldus Information Technology Inc., Wageningen, The Netherlands). Coding of videos was done blind to both the model and playback type to which the bird had been assigned, by a single observer (G.M.). The number of flights made by the bird in the 60 s prior to the model presentation, and 60 s following the presentation were recorded, as was the distance travelled during these flights. There was a high level of collinearity between the number of flights and distance travelled (*r* = 0.964), so the number of flights made was used as a response variable as this was less subjective. We also recorded any vocalizations made by the birds. We analysed the data using R version 3.1.2 (R Core Team), and the packages *lme4*, *pastecs*, *ggplot2* for graphs, and *lmtest* for Breusch-Pagan tests.

We analysed the influence of the presentation number (1, 2 or 3), model (fox or elephant), and playback type (scold or contact calls) on the number of flights that the birds made in the 60 s after each presentation, which we interpreted as an indicator of individuals' stress/escape responses towards the model. We used general linear mixed models (GLMMs; *lme4* package), with focal bird identity and playback track fitted as random effects. The number of flights made by the birds in the 60 s before each presentation was included as an additional explanatory term in each model, to account for any captivity-induced agitation and/or carry-over effects from previous presentations. Over-dispersion in the raw data made Poisson error structure unsuitable, so we square-root transformed the response variable (number of flights in 60 s post-presentation) which allowed the model to be fitted with a Gaussian error structure. Model plots were examined for evidence of violation of assumptions, and refitted as general linear models to allow a Breusch-Pagan test to check for heteroscedasticity in the data. We fitted 19 models in total, containing all potential combinations of the three main explanatory variables (model, playback type and presentation number) and the possible interaction terms, as well as the number of flights in the 60 s prior to the presentation as a main effect only.

To further examine the factors influencing responses in the test phase, we conducted an additional analysis, using only the data from the 60 s before and after presentation 3. In this analysis we used linear regression models, to test the influence of the playback, model, and the number of flights made in the 60 s prior to presentation 3 on the number of flights made by the birds in the 60 s after. The number of flights before and after the presentation was square-root transformed, as in the previous analyses.

We used an information theoretic (IT) approach to model selection, using Akaike's information criterion corrected for small sample sizes (AICc) to rank the models, following the approach advocated by Richards *et al*. [35]. Models that had a ΔAICc ≤ 6 of the model with the lowest AICc value (table 1) formed the ‘top set’. We then applied the ‘nesting rule’ [36] to the top set, whereby models that were more complex versions of nested models with a lower AICc value were removed from the top set so as not to retain unnecessarily complex models.

**Table 1.** Model selection table for the variables influencing the (square-root transformed) number of flights made by the jackdaws in the 60 s after each presentation. (The italics highlights the models that form the top set prior implementation of a model nesting rule [36] that filtered out those that should not be retained. When factors are included in the model this is denoted by the symbol —, and numbers refer to the coefficients of numeric variables when these were included in the model, while an asterisk denotes interaction terms between variables. sqrt.pre60 refers to the number of flights made in the 60 s prior to the presentation (square-root transformed), while model denotes the model shown (elephant/fox), pres.num the presentation number (1, 2 or 3), and pb.group whether the bird heard scold calls or contact calls during presentation 2. Potential ‘top’ models are highlighted in bold, and these are reported in full in table 2.)

model ID	intercept	sqrt.pre60	model	pb.group	pres.num	model* pb.group	model* pres.num	pb.group* pres.num	model* pb.group* pres.num	df	logLik	AICc	ΔAICc	retained	weight
***sqrt.sel6***	***1.815***	***0.521***	**—**	**—**	**—**		**—**			***11***	**−*213.8***	***451.5***	***0***.***0***	***yes***	***0.53***
***sqrt.sel9***	***1.991***	***0.527***	**—**		**—**		**—**			***10***	**−*215.1***	***451.8***	***0***.***3***	***yes***	***0.47***
*sqrt.sel3*	*1.753*	*0.527*	**—**	**—**	**—**	**—**	**—**			*12*	−*213.6*	*453.7*	*2*.*1*	*no*	
*sqrt.sel5*	*1.943*	*0.508*	**—**	**—**	**—**		**—**	**—**		*13*	−*213.3*	*455.4*	*3*.*9*	*no*	
sqrt.sel2	1.882	0.514	**—**	**—**	**—**	**—**	**—**	**—**		14	−213.2	457.6	6.1		
sqrt.sel15	1.679	0.526		**—**	**—**					8	−220.8	458.7	7.1		
sqrt.sel18	1.852	0.532			**—**					7	−222.1	459.0	7.5		
sqrt.sel12	1.637	0.522	**—**	**—**	**—**					9	−220.7	460.7	9.2		
sqrt.sel14	1.812	0.528	**—**		**—**					8	−222.0	461.1	9.6		
sqrt.sel1	1.869	0.504	**—**	**—**	**—**	**—**	**—**	**—**	**—**	16	−212.6	461.5	10.0		
sqrt.sel11	1.807	0.514		**—**	**—**			**—**		10	−220.4	462.4	10.9		
sqrt.sel7	1.573	0.529	**—**	**—**	**—**	**—**				10	−220.6	462.8	11.3		
sqrt.sel8	1.764	0.509	**—**	**—**	**—**			**—**		11	−220.3	464.6	13.1		
sqrt.sel17	1.472	0.525		**—**						6	−226.1	464.9	13.4		
sqrt.sel19	1.644	0.532								5	−227.4	465.3	13.8		
sqrt.sel4	1.701	0.516	**—**	**—**	**—**	**—**		**—**		12	−220.2	466.8	15.2		
sqrt.sel13	1.431	0.521	**—**	**—**						7	−226.1	466.9	15.4		
sqrt.sel16	1.605	0.528	**—**							6	−227.4	467.3	15.8		
sqrt.sel10	1.366	0.528	**—**	**—**		**—**				8	−225.9	468.9	17.4		

## 3. Results

### 3.1. Effects of training on responses

**Figure 1.**
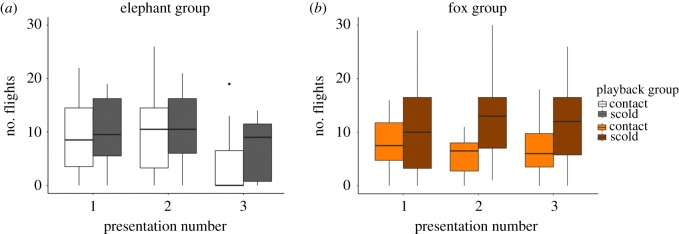
Plots of the raw data for the number of flights made by the birds in the 60 s following the presentation of (*a*) the model elephant, or (*b*) the model fox. Light colours in each represent birds from the contact call group, while the darker plots display data from birds played scold calls in presentation 2. There were no accompanying playbacks in presentations 1 and 3.

Individuals showed substantial variation in their responses to the three model (i.e. fox/elephant) presentations (electronic supplementary material, figure S3). In mixed model analysis, four models formed the top set (table 1), and from these models numbers 6 and 9 were retained (table 2) following application of the nesting rule [36]. In both models, the number of flights made in the 60 s prior to each presentation being made was a strong positive predictor of how many flights were made in the 60 s following the presentation (table 2). Both candidate models also included an interaction between presentation number and model type. Birds shown the elephant model displayed a substantial decrease in response to presentation 3 compared to presentation 1, while those shown the fox did not ($\bar{x\;}$(s.e.) number of flights made in the 60 s after presentation: fox presentation 1 = 9.66 (1.59), fox presentation 3 = 9.46 (1.41); elephant presentation 1 = 9.75 (1.41), elephant presentation 3 = 5.75 (1.28); figure 1, table 2).

**Table 2.** Values from the GLMM models highlighted in table 1 as the being the candidate ‘best’ models for predicting which factors influenced the (square-root transformed) number of flights made by the birds around the experimental arena in the 60 s after the model presentation. (Bird identity and playback heard were included as random effects in each model, with the variance (s.d.) of bird identity being 0.303 (0.55) in model 6 and 0.331 (0.58) in model 9. Variance attributable to the playback track was zero in all models.)

variable	estimate	s.e.	*t*-value	*p*-value
*model sqrt.sel6 summary*				
intercept	1.815	0.264	6.87	<0.001
sqrt. flights in 60 s prior to pres	0.521	0.064	8.16	<0.001
model				
elephant	0	0		
fox	−0.257	0.318	−0.81	0.42
playback group				
contact	0	0		
scold	0.369	0.226	1.63	0.11
presentation number				
pres 1	0	0		
pres 2	0.067	0.275	0.25	0.81
pres 3	−1.221	0.275	−4.43	<0.001
presentation number × model	0			
pres 2:fox	−0.154	0.389	−0.40	0.693
pres 3:fox	1.216	0.389	3.13	0.002
*model sqrt.sel9 summary*				
intercept	1.991	0.247	8.07	<0.001
sqrt. flights in 60 s prior to pres	0.527	0.064	8.22	<0.001
model				
elephant	0	0		
fox	−0.26	0.322	−0.81	0.42
presentation number				
pres 1	0	0		
pres 2	0.068	0.275	0.25	0.81
pres 3	−1.223	0.276	−4.43	<0.001
presentation number × model				
pres 2:fox	−0.156	0.389	−0.40	0.69
pres 3:fox	1.217	0.389	3.13	0.002

Between presentation 1 and presentation 2 there was no change in the response of the birds to the presentation, regardless of the model shown or playback heard in presentation 2, with the birds showing a similar level of alarm in each (table 2, figure 1). Birds that were played scolds in presentation 2 generally flew more in response to presentations across all presentation periods and regardless of the model shown ($\bar{x\;}$(s.e.) number of flights made in the 60 s after presentation: contact call group birds = 7.42 (0.76); scold call group birds = 10.56 (0.89)). However, while playback type was a term in the model with the lowest AICc (model 6; table 1), it did not appear to have a robust effect on the number of flights made in the 60 s after the presentation (estimate (s.e.) = 0.369 (0.226), *t* = 1.63, *p* = 0.11; model 6; table 2) and did not appear in the second best model (model 9; table 1).

Only two birds made scold calls during the experiment, and neither case occurred during the 60 s after a model presentation. Both cases occurred between presentations 2 and 3, with one bird (fox-scold group) directing the vocalization at a passing buzzard (*Buteo buteo*), while the other (fox-contact group) appeared to direct the vocalization in the direction of the hide where the fox was concealed.

### 3.2. Potential carry-over effects of playbacks

**Figure 2.**
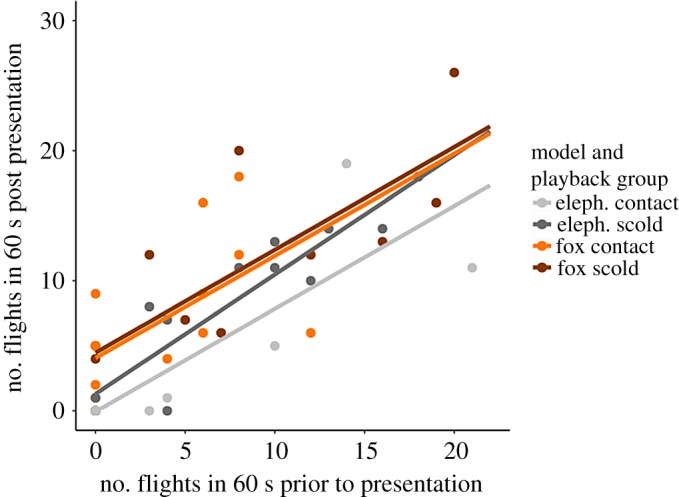
Scatterplot of the number of flights made in the 60 s prior to presentation 3, against the number of flights made in the 60 s after the presentation, highlighting the consistency of this relationship between groups, regardless of the model shown or the playback previously heard.

In the analysis that considered only the responses to the final, *test* presentation, there were three models in the top set, but after the nesting rule was applied only one was retained (p3.model4) (table 3, electronic supplementary materials, S2). As in the previous analysis, birds were likely to fly more in response to presentations of the fox than the elephant (estimate (s.e.) = 0.916 (0.245), *t* = 3.74, *p* < 0.001; figure 1) and the number of flights made in the 60 s pre-presentation predicted the number of flights after the presentation (estimate (s.e.) = 0.777 (0.077), *t* = 10.06, *p* < 0.001; figure 2). The number of flights in the 60 s prior to the presentation (pre60) was by far the best predictor of post-presentation responses, with all models containing this term having an adjusted *R*^2^ ranging from 0.63 to 0.72 (table 3). However, if we did not account for agitation, and left pre60 out of the analysis, then playback type changed from being a non-significant predictor of the birds' behaviour following presentation 3 to having *α* ≤ 0.05 (p3.model6 and p3.model9). On average, birds that had been played scolds in presentation 2 flew more than those that had heard contact calls ($\bar{x\;}$(s.e.) number of flights made in the 60 s after presentation: scold call group = 9.5 (1.4), contact call group = 5.7 (1.3); estimate (s.e.) = 0.886 (0.421), *t* = 2.10, *p* = 0.04).

**Table 3.** Model selection table for the variables influencing the (square-root transformed) number of flights made by the jackdaws in the 60 s after presentation 3 only. (The italics highlights the models that form the top set prior implementation of a model nesting rule [36] that filtered out those that should not be retained. When factors are included in the model this is denoted by the symbol —, and numbers refer to the coefficients of numeric variables when these were included in the model, while an asterisk denotes interaction terms between variables. sqrt.pre60 refers to the (square-root transformed) number of flights made in the 60 s prior to the model presentation, while model denotes the model shown (elephant/fox), and pb.group whether the bird heard scold calls or contact calls during presentation 2. The top model is highlighted in bold. As there was only one top model following application of the nesting rule, model weights are provided for all of the models listed.)

model.ID	intercept	sqrt.pre60	model	pb.group	model* pb.group	d.f.	logLik	AICc	ΔAICc	weight	retained	*R*^2^	adj-R^2^
***p3.model4***	***0.305***	***0.777***	**—**			***4***	**−*58.7***	***126.2***	***0***.***0***	***0.50***	***yes***	***0***.***73***	***0.71***
*p3.model2*	*0.201*	*0.754*	—	—		*5*	−*57.9*	*127.3*	*1.1*	*0.30*	*no*	*0.73*	*0.72*
*p3.model1*	*0.036*	*0.760*	—	—	—	*6*	−*57.0*	*128.1*	*1.9*	*0.20*	*no*	*0.74*	*0.72*
p3.model7	0.733	0.792				3	−65.1	136.8	10.6	0.00		0.64	0.63
p3.model3	0.636	0.770		—		4	−64.6	138.2	12.0	0.00		0.65	0.63
p3.model6	1.302		—	—		4	−84.7	178.3	52.1	0.00		0.19	0.15
p3.model8	1.745		—			3	−87.0	180.5	54.2	0.00		0.11	0.09
p3.model5	1.210		—	—	—	5	−84.6	180.6	54.4	0.00		0.19	0.14
p3.model9	1.824			—		3	−87.8	182.1	55.8	0.00		0.08	0.06

## 4. Discussion

Predation is typically the main cause of mortality for fledgling birds [37–39], so there is great pressure on young individuals to quickly learn how to identify and respond to potential predators. While experiments have demonstrated that birds can learn about novel predators through exposure to social information over several minutes [19,23], it remains unclear whether the very brief exposures to social information that often accompany natural predator attacks are sufficient to promote learning. Moreover, experimental tests of anti-predator social learning outside of controlled laboratory settings remain rare.

Here we found that, contrary to expectations, brief exposures to social information in the form of conspecific vocalizations did not appear to produce a consistent change in young jackdaws’ responses to potential predators. If social learning had a strong effect, we would expect naive birds to show low baseline responses to models which increased following scold call training and declined or remained constant following control call training. Instead, birds often showed high levels of agitation from the start of the experiment, with an overall decline in the number of flights made in response to the elephant from presentation 1 to presentation 3, while responses to the fox remained elevated throughout, regardless of training. This suggests that the jackdaws may have habituated to the novel elephant stimulus through repeated presentations, and that most if not all individuals already recognized the fox as a threat from the start of the experiment. Although visual inspection of figure 1 suggests that the responses of birds given scold call training may have remained elevated while those with contact call training declined, our analyses showed no clear statistical evidence for an interaction between presentation number and playback type. We did find some weak support for a main effect of playback type, with scold-trained birds tending to fly more than those that heard contact calls (playback type features in the top model, but does not have a significant effect at *α* = 0.05). However, this effect was consistent across both model groups, suggesting that it could be the result of birds still being in an elevated state of agitation as a result of recently having heard scold calls, rather than specific learned responses towards the models. Indeed, the number of flights made in the minute before presentations was by far the strongest predictor of the subsequent responses to the presentations (figure 2), indicating that captivity- and/or carry-over induced agitation rather than social learning was the primary driver of behaviour.

This conclusion is further supported by analyses honing in on responses to the final, test phase. Here, as in the main analysis, we found stronger responses to the fox than to the elephant but, as before, the strongest predictor was the number of flights in the minute preceding the presentation. Playback type did not appear to have a robust effect on responses, as it did not feature in the top model, and its inclusion produced only a 1% improvement in the variance explained (adjusted *R*^2^ of model 2 = 0.72; model 4 = 0.71). To the extent that there was some small increase in the responses of birds that heard scold calls compared to those that heard contact calls, this was consistent across both model types and is most likely to represent a carry-over effect from calls heard in training. It is important to note that, had we not accounted for levels of agitation prior to presentations, we could have reached very different conclusions, as playback type did appear to have a statistically significant effect (*p* < 0.05) when pre-presentation flights were not included in the analysis.

Although our experiment failed to generate the predicted effects, it nevertheless has a number of important fundamental and practical implications. First, our results show that taking levels of agitation induced by captivity and/or carry-over effects into account is critical for studies attempting to train animals to respond to novel stimuli. Given the growing emphasis on harnessing social learning to promote adaptive behaviours in conservation contexts [40,41], accounting for agitation will be vital in the design and interpretation of such research. This may be particularly important for species such as corvids that exhibit high levels of neophobia [42,43] and for *in situ* conservation and wildlife management schemes, where animals are not used to captive conditions. More generally, a failure to consider captivity-induced agitation could lead to false conclusions in any behavioural assay where animals are brought into captivity. For instance, some studies have interpreted movement around an enclosed space as indicative of exploratory tendencies, when it may in fact reflect agitation induced by spatial neophobia (reviewed in [44]) and/or elevated stress levels owing to capture and handling [45]. The high flight responses observed in response to the first baseline presentation, regardless of the model shown, suggests that at this is likely to be the case here. While levels of agitation could, in principle, be reduced in experiments such as ours by allowing subjects more time to acclimatize to captive conditions, this must always be traded off against the ethical imperative to keep presentations as short as possible, particularly if they involve young animals that are still dependent on parents.

Second, our experiment showed that responses differed substantially between individuals (evident in the electronic supplementary material, figure S3). While such differences might be influenced by previous experience with predators [46,47], it is also possible that individual differences in agitation (possibly linked to variation in stress reactivity or coping styles; [48]) could affect the potential for individuals to learn about novel predators, both in experimental and natural contexts. While a hormonally mediated stress response is known to be necessary to promote aversion learning [49,50], there is some evidence that acute levels of stress can inhibit learning [51]. Moreover, highly anxious individuals may be faster to flee upon hearing conspecific alarm calls, thus reducing their potential for learning to associate the calls with the presence of a novel predator. Given evidence that personality differences influence social information use in other contexts (e.g. [52,53]), understanding how personality affects the development of anti-predator responses is a clear priority for future research.

Finally, our results raise questions about the stimuli needed to promote social learning of anti-predator responses. Whereas some studies on captive animals show that playbacks of mobbing or alarm calls are sufficient to promote learned fear responses towards novel stimuli [18,24], this did not appear to be the case in our experiment. Indeed, as the great majority of research on socially learned anti-predator responses has taken place under controlled laboratory conditions, it remains unclear whether vocalizations alone are sufficient to promote learning in the wild. Corvids, for example, show a range of distinctive behaviours in response to predators, including gaze fixing, aggressive postures and diving flights directed at the threat [54–56]. It is quite possible that the posture and directedness of the behaviour of conspecifics is as important as, or complimentary to, the vocalizations themselves in serving to reinforce learning and increase signal saliency. Such multimodal signals have been found to increase the speed at which predators learn to discriminate aposematic prey [57], and also enhance the responses of observers to anti-predator alarm signals compared to when such signals are presented singly [58].

Understanding how wild animals learn about new threats is an important priority for both fundamental and applied research. Tightly controlled laboratory experiments show that anti-predator responses can, in principle, be socially learned, but work on wild animals remains rare. We must now embrace the complexities of the real world, including individual variation and multimodal signal structure, to better understand how such learning operates in practice.

## Supplementary Material

Supp Mat - McIvor et al - Social learning of threats and captivity induced agitation

## Data accessibility

The data and R code are available from the Dryad Digital Repository at http://doi.org/10.5061/dryad.9r8v0 [60].
