# Supplementary material for: Testing social learning of anti-predator responses in juvenile jackdaws: the importance of accounting for levels of agitation
Source: R Soc Open Sci. 2018 Jan 24;5(1):171571. doi: 10.1098/rsos.171571 (PMC5792938; doi:10.1098/rsos.171571)

**Supplementary Material**

**S1:** Diagram demonstrating the layout of the experimental arena. 1) is the hide containing the experimenter, concealed before the bird was released, 2) is the hide containing the model, with 3) indicating the rubble pile that obscured the skateboard from the view of the birds. 4) Is the experimental aviary. The three speakers (5) were arrayed around the hide, 5 metres apart. Perches are shown by the brown bars, and the two screens by the brown squares. Food and water bowls are represented by the circles. The right half of the aviary was roofed to provide shelter from the elements. There was a 10m gap between the hides and the aviary when the experiment was conducted. The gap shown here is not to scale.

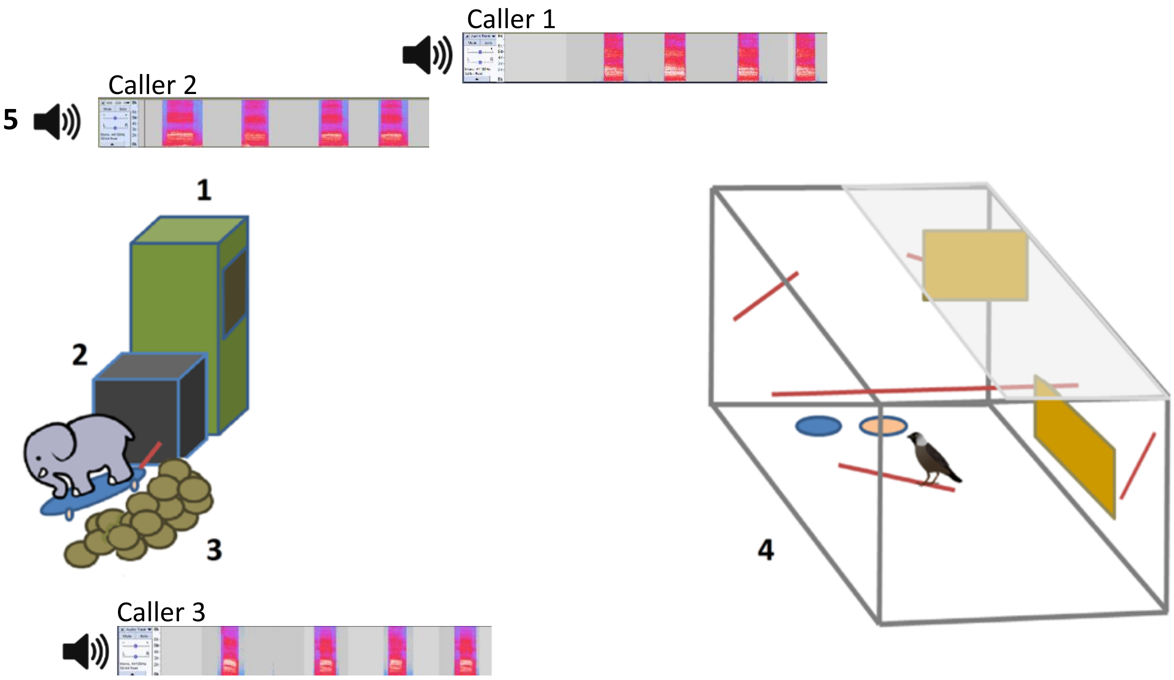


**S2:** Model summary for linear model p3.model4. This was the only retained model from the analysis of which factors influenced the (square-root transformed) number of flights made by the birds around the experimental arena in the sixty seconds after the third model presentation was made.

| Variable | Estimate | SE | t-Value | P-Value |
|---|---|---|---|---|
| Intercept | 0.305 | 0.225 | 1.36 | 0.18 |
| sqrt.Flights in 60s prior to Pres | 0.777 | 0.077 | 10.06 | <0.001 |
| Model: Elephant | 0 | 0 | | |
| Fox | 0.916 | 0.245 | 3.74 | <0.001 |

**S3:** Line graphs plotting the changes in response to the experimental stimulus of each individual bird across the three presentation periods for those shown the Elephant model (a), and those shown the Fox (b). Birds in the contact call group are shown in the lighter colour on each graph.

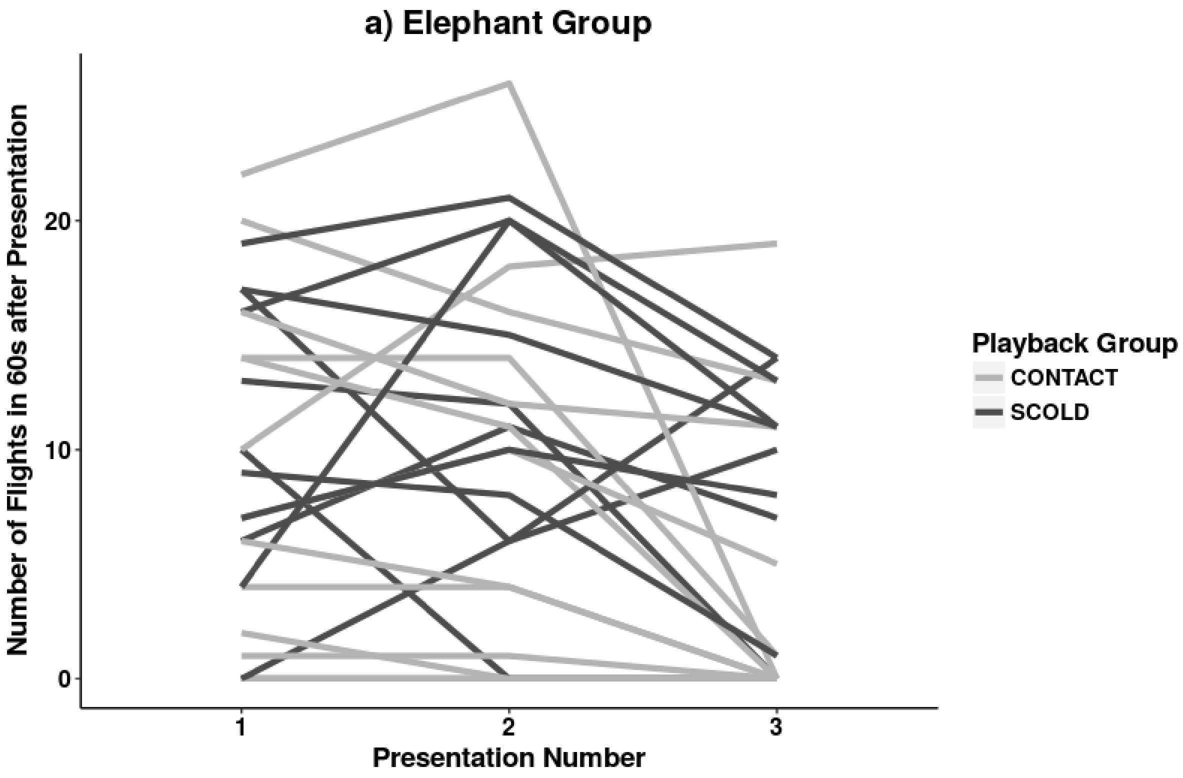


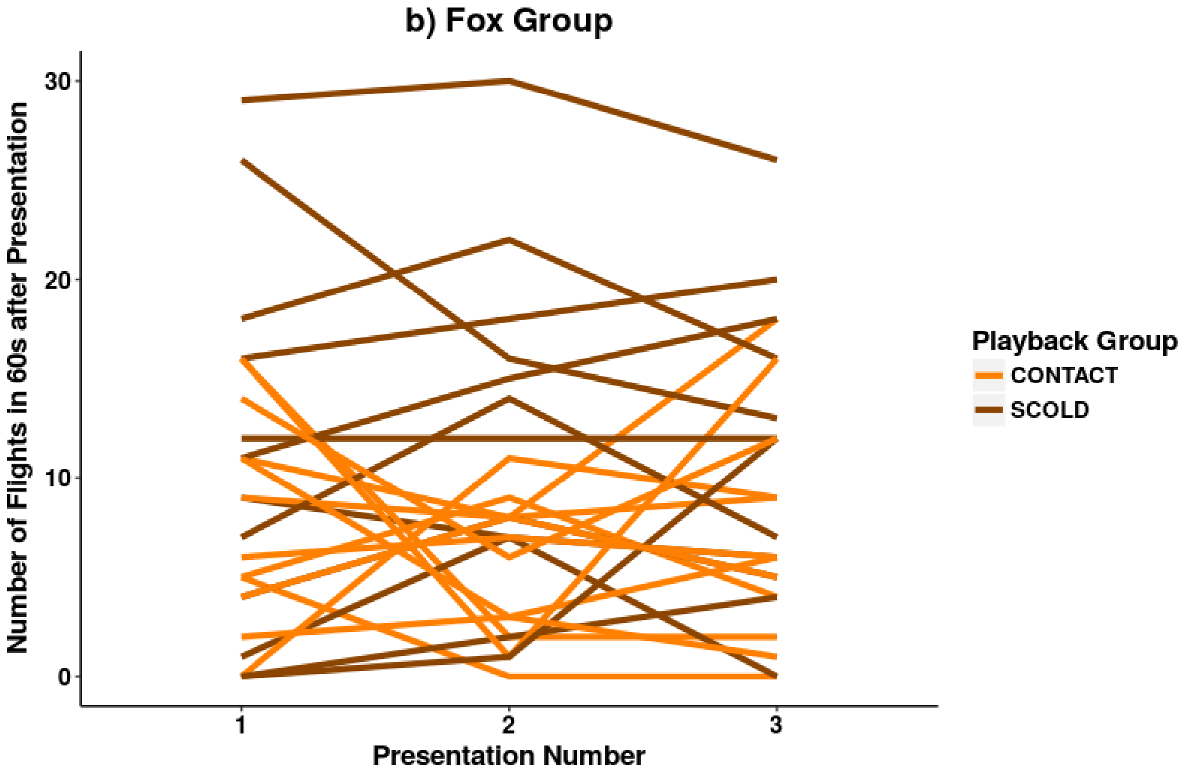

Supplement: Supp Mat - McIvor et al - Social learning of threats and captivity induced agitation [file rsos171571supp1.pdf]
